# Surface structure promoted high-yield growth and magnetotransport properties of Bi_2_Se_3_ nanoribbons

**DOI:** 10.1038/s41598-019-47547-0

**Published:** 2019-08-05

**Authors:** Gunta Kunakova, Raimonds Meija, Jana Andzane, Uldis Malinovskis, Gvido Petersons, Margarita Baitimirova, Mikhael Bechelany, Thilo Bauch, Floriana Lombardi, Donats Erts

**Affiliations:** 1 Institute of Chemical Physics, University of Latvia, Raina blvd 19, Riga, LV-1856 Latvia; 2 European Institute of Membranes, University of Montpellier, CNRS, ENSCM, 34095 Montpellier, France; 3 Department of Microtechnology and Nanoscience, Chalmers University of Technology, SE-41296 Gothenburg, Sweden; 4 Faculty of Chemistry, University of Latvia, Raina blvd 19, Riga, LV-1856 Latvia

**Keywords:** Synthesis and processing, Topological insulators, Topological insulators, Electronic devices

## Abstract

In the present work, a catalyst-free physical vapour deposition method is used to synthesize high yield of Bi_2_Se_3_ nanoribbons. By replacing standard glass or quartz substrates with aluminium covered with ultrathin porous anodized aluminium oxide (AAO), the number of synthesized nanoribbons per unit area can be increased by 20–100 times. The mechanisms of formation and yield of the nanoribbons synthesized on AAO substrates having different arrangement and size of pores are analysed and discussed. It is shown that the yield and average length of the nanoribbons can base tuned by adjustment of the synthesis parameters. Analysis of magnetotransport measurements for the individual Bi_2_Se_3_ nanoribbons transferred on a Si/SiO_2_ substrate show the presence of three different populations of charge carriers, originating from the Dirac surface states, bulk carriers and carriers from a trivial 2DEG from an accumulation layer at the Bi_2_Se_3_ nanoribbon interface with the substrate.

## Introduction

Bismuth selenide (Bi_2_Se_3_) is a narrow bang gap layered semiconductor, previously widely studied as one of the best materials for near room-temperature thermoelectrical applications^1^. The discovery that this material belongs to a new class of quantum matter – three-dimensional topological insulators (TIs) possessing topological surface states, protected by the time-reversal symmetry due to the strong spin-orbit coupling^2,3^, entailed a new wave of research on this material.

The TI properties of Bi_2_Se_3_ have been mostly investigated by measurements on cleaved surfaces of bulk crystals^4^ and molecular beam epitaxy (MBE) synthesized thin films^5^. It was realised, that the residual (native) doping arising from the Se vacancies in as-synthesized Bi_2_Se_3_^6^ is the main obstacle for a direct investigation of the fundamental properties of topological surface states, and requires additional treatment to minimize dominance of the bulk transport. A promising approach to access the surface effects of the TIs is to reduce the size of material down to the nanoscale, thus increasing the surface to volume ratio. Nanostructures prepared by different techniques, such as chemical vapour deposition (CVD)^7^, sonochemical^8^ and solvothermal^9^ methods, catalysed vapour-liquid-solid^3,10,11^ and catalyst-free physical-vapour deposition (PVD)^12–14^ have been studied. However, most of the listed techniques have drawbacks associated with surface contamination of the nanostructures due to catalyst residues or low-crystalline structure due to the low process temperatures.

The catalyst-free PVD technique is especially attractive for the fundamental investigations of exotic properties of topological surface states. The nanoribbons produced by the catalyst-free PVD method on glass substrates^13,14^ showed high-crystalline structure, as well as very high charge carrier mobilities up to 8000 cm^2^/Vs^13^. It was shown that by reducing the thickness of the nanoribbon it is possible to supress the charge carriers from the bulk of the nanoribbon, thus entering the *bulk – free* conduction regime^14^.

The high Young’s modulus (44 GPa^15^) and low resistivity of the Bi_2_Se_3_ nanoribbons makes these nanostructures perspective also for applications in nanoelectromechanical switches^16,17^.

A drawback of the nanoribbon fabrication by catalyst-free PVD technique is the relatively low yield of the nanoribbons. This hampers the use of catalyst-free PVD method for mass-production of the nanoribbons, required by some applications, as for example, nanoribbon-containing solution for the dielectrophoretic alignment of the nanoribbons. Previously, a high yield growth of the Bi_2_Se_3_ nanoribbons by PVD was demonstrated using Au nanoparticles as a catalyst^18^ and indium tin oxide (ITO) covered glass substrates^16^. However, the nanoribbons grown by these methods may be doped by catalyst atoms. This may result in increased bulk carrier density screening the transport properties from the topological surface states.

Previously, the AAO membranes have proven to be ideal substrates for the production of nanowire arrays using electrochemical, supercritical fluid and CVD-based techniques^19–23^, as well as for sorting and ordering of nanoparticles on the surface of AAO membranes^24,25^, and enhancement of plasmonic scattering in structured Al-AAO-Au layers^26^. In this work, porous anodized aluminium oxide (AAO) substrates are used for catalyst-free PVD synthesis of Bi_2_Se_3_ nanoribbons. The results of our work show that the use of AAO substrates is perspective for a high-yield fabrication of the *bulk – free* Bi_2_Se_3_ nanoribbons. The mechanisms of the nucleation and growth of the nanoribbons on the AAO substrates prepared by different techniques are discussed.

To characterize the charge transport in nanoribbons grown on AAO, a set of magnetotransport measurements for individual nanoribbons have been performed. By combining gate dependent Shubnikov de Haas (SdH) oscillation measurements and Hall effect characteristics, carriers from different charge carrier bands are identified contributing to the total charge carrier density: carriers from the Dirac surface states, bulk carriers and the carriers from a trivial 2DEG from an accumulation layer at the interface of the nanoribbon and the substrate.

## Results and Discussion

### Nucleation, growth conditions, structure and dimensions of Bi_2_Se_3_ nanoribbons

**Figure 1:**
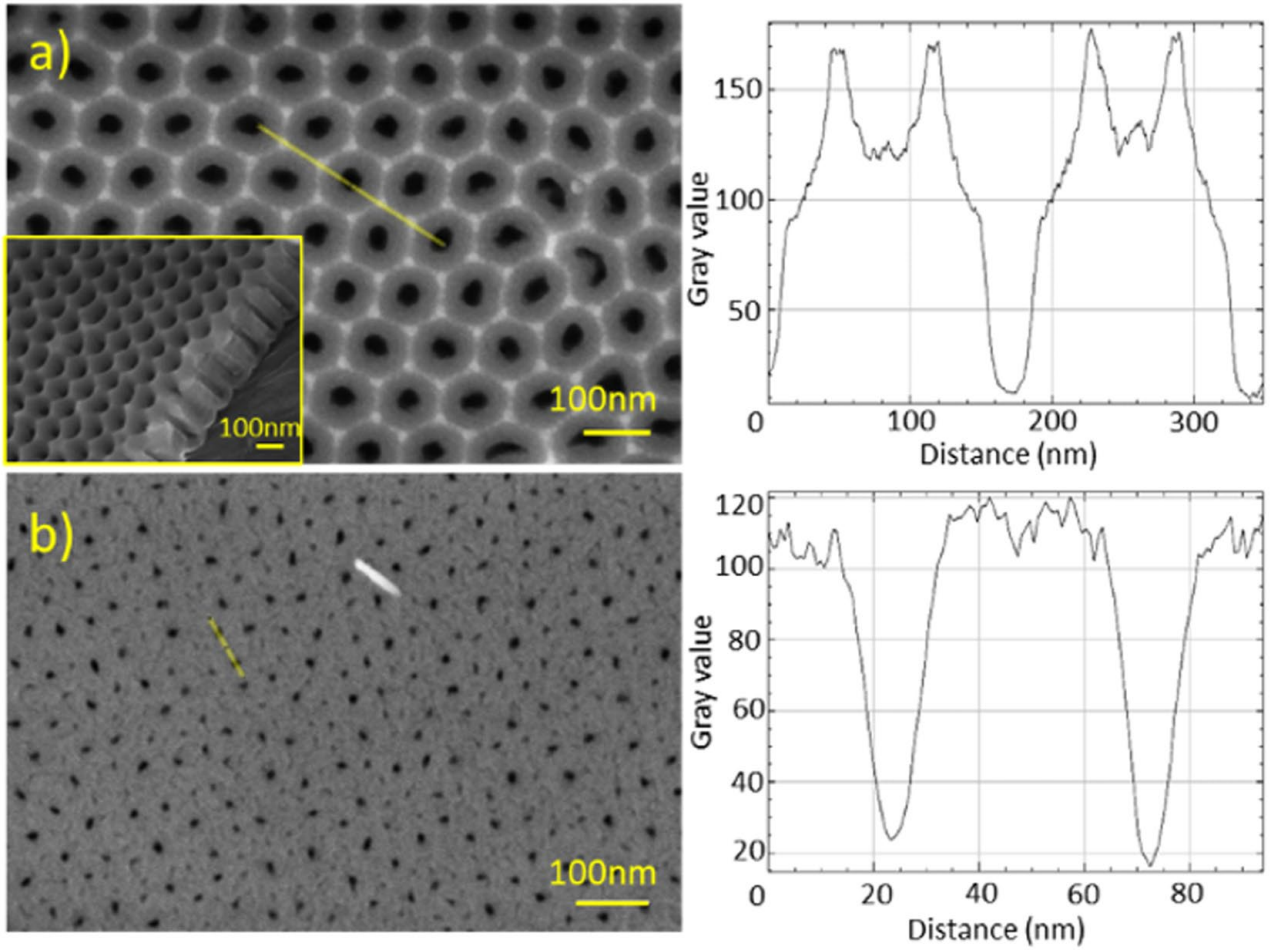
SEM images of ultrathin AAO films: (**a**) - top and tilted view of a film with ordered pore diameter 41 nm fabricated in the second anodization step in oxalic acid; (**b**) - top view of a film with nonordered pore diameter of 15 nm fabricated in the first anodization step in sulphuric acid. Corresponding surface plots are shown in the right panels.

To analyse the results of nanoribbon growth on different surfaces, the Bi_2_Se_3_ nanostructures grown on a glass substrate were compared to the nanostructures grown on 3 different anodized AAO surfaces of similar thicknesses (250–300 nm) (Fig. 1 inset) but different pore diameters and topography of interpore junctions (Fig. 1a,b).

For the ultrathin films with regular pore arrangement fabricated in a second anodization step with a mean pore diameter of 41 ± 6 nm (anodized at 40 V in oxalic acid, Fig. 1a) and 24 ± 4 nm (anodized at 25 V in sulphuric acid), funnel type pore openings and oxide asperities at three pore contact points are clearly seen (Fig. 1a, top right panel). However, AAO membranes with disordered pore arrangement fabricated in the first anodization step contain pores (mean pore diameter 15 ± 3 nm) without funnel type openings and asperities between them (Fig. 1b, bottom right panel). Ordered AAO surface also have a larger surface area compared to the disordered AAO surface and glass thus providing larger surface areas for the possible nucleation of Bi_2_Se_3_ crystals.

**Figure 2:**
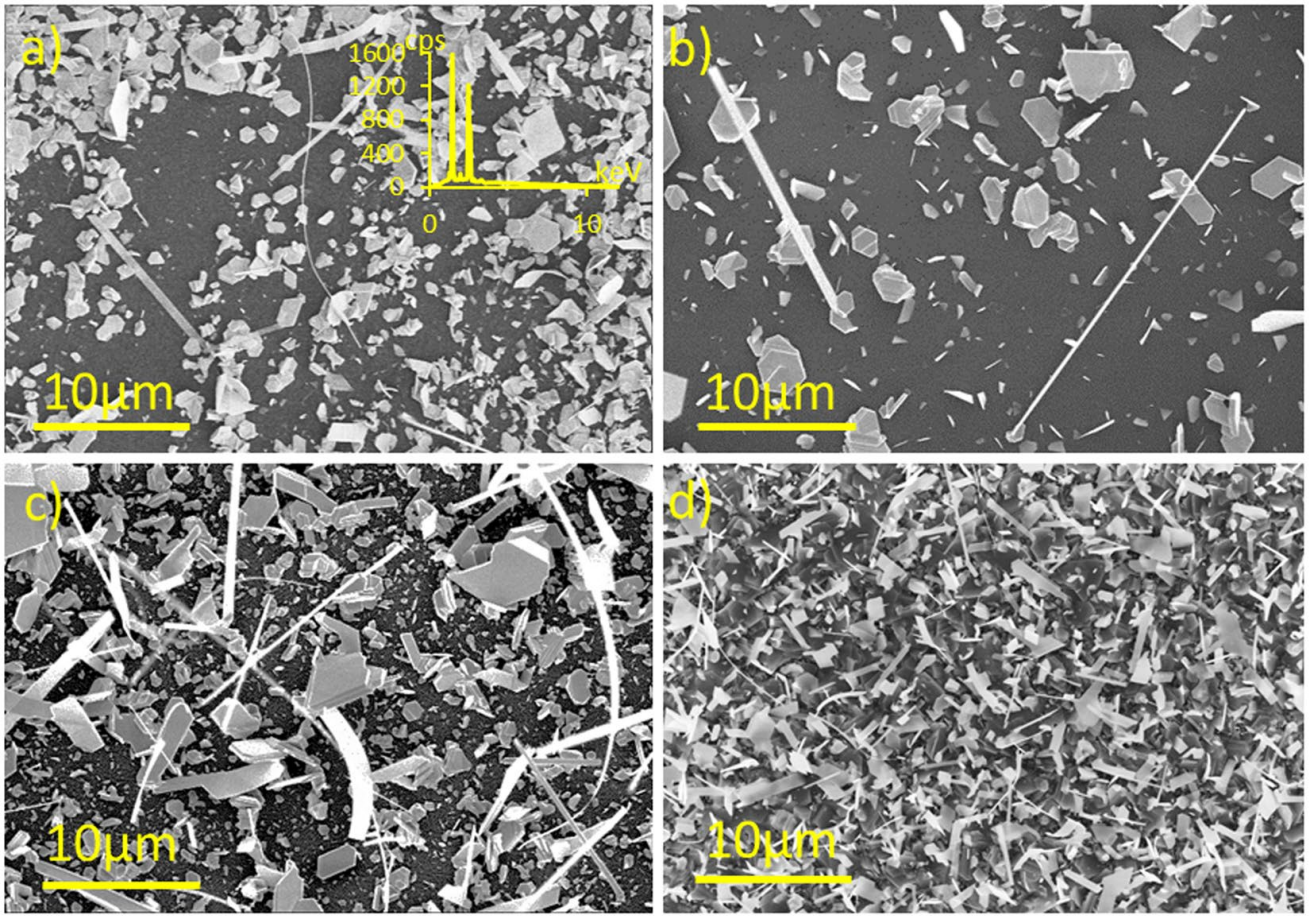
SEM images of Bi_2_Se_3_ nanostructures on: (**a**) – glass (inset – EDX spectra of Bi_2_Se_3_ nanoribbons deposited on fused quartz surface); (**b**) – AAO, average disordered pore size 15 nm; (**c**) – AAO, average ordered pore size 41 nm; (**a**–**c**) synthesis parameters: source material heating rate 12.5 °C/min, start-end pressure 5–20 Torr; (**d**) – AAO, average ordered pore size 24 nm, synthesis parameters: source material heating rate 18.5 °C/min, start-end pressure 0.5–4.5 Torr).

Estimation from the low magnification SEM images of the Bi_2_Se_3_ nanostructures grown on glass and AAO surfaces (Fig. 2) revealed that the number of the nanoribbons per unit area grown on the AAO surfaces where nanopores have distinct asperities between them (Fig. 1a right panel, Fig. 2c,d), is 20–100 times higher in comparison to the AAO substrates with no distinct asperities (Fig. 1b right panel, Fig. 2a) and the glass substrates (Fig. 2b).

EDX data (Fig. 2a – inset) prove that the grown Bi_2_Se_3_ nanostructures are stoichiometric – ratio of Bi and Se corresponds to 40% Bi: 60% Se for all the structures grown on glass and AAO substrates.

Increase in the number of the nanostructures and nanoribbons grown on the AAO substrates with ordered pore structures in comparison to the nanostructures grown on the glass surface cannot be explained only by the presence of the pores. The synthesis of the nanoribbons on the AAO substrates with the disordered pores of size 15 nm showed the yield of the nanoribbons comparable with the one obtained on the glass substrate (Fig. 2a,b).

**Figure 3:**
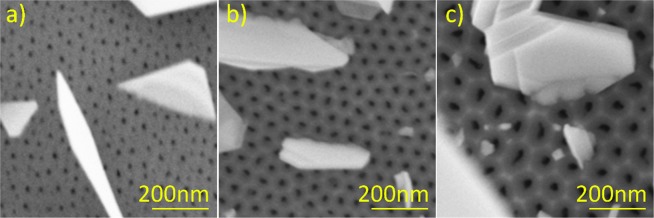
Initial growth of Bi_2_Se_3_ nanoplates on different AAO substrates: (**a**) – 15 nm disordered pores; (**b**) –24 nm ordered pores; (**c**) – 41 nm ordered pores.

Presumably, the high yield of the nanoribbons may be related to the increased number of tilted Bi_2_Se_3_ nanoplates grown on the AAO substrates with pronounced nanoporous structure. As this has been shown previously, the nanoribbons start to grow from the seed nanoplates^12^. The surfaces with ordered nanoporous structure may promote growth of Bi_2_Se_3_ nanoplates, tilted under different angles relative to the substrate basis (Fig. 3a–c).

The Bi_2_Se_3_ crystals start their growth as isolated nanoplatelets (like Volmer-Weber mode^27^) presumably on the funnel-like pore openings or asperities of an ordered AAO surface. According to the island type crystal growth model, Bi and Se atoms are more strongly bound to each other than to the AAO substrate, and the surface asperities may serve as energetically favourable adsorption sites. Similar nucleation of ZnO crystals on open ends of the pores of the AAO template was observed by the other group^28^. It should be noted that although the AAO membranes during their fabrication process obtain sulphur, oxalic and phosphate residues, it is unlikely that they are prominent nucleation centres for Bi_2_Se_3_ nanoplate growth.

**Figure 4:**
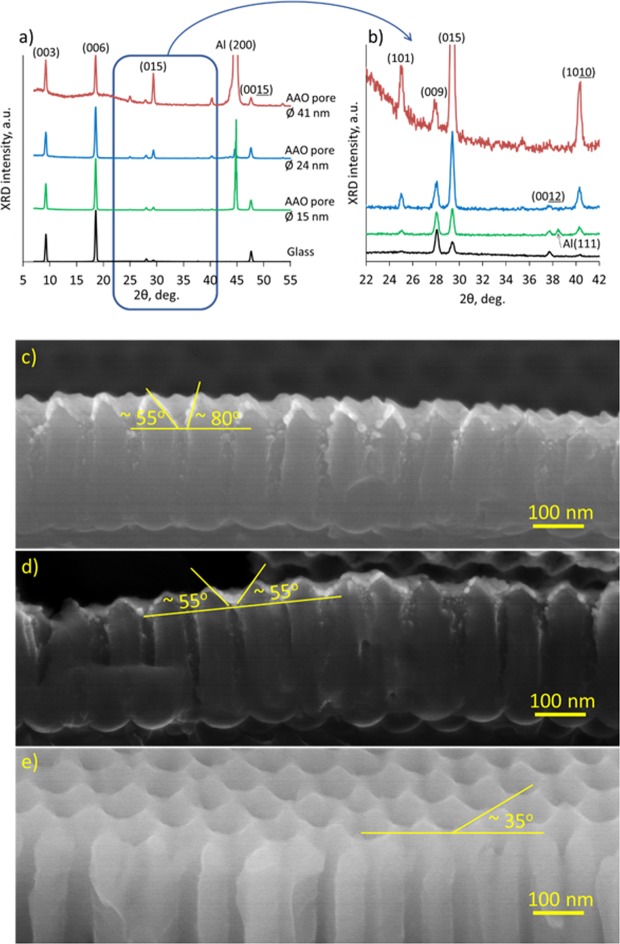
(**a**) XRD spectra of Bi_2_Se_3_ grown on glass and on AAO with average disordered pore size 15 nm; average ordered pore size 24 nm and average ordered pore size 41 nm; (**b**) closer look at the XRD diffraction peaks corresponding to the tilted Bi_2_Se_3_ nanoplates; (**c**–**e**) side-view SEM images of the AAO substrates, illustrating the slope angles of the nanopore openings.

The XRD spectra obtained for the Bi_2_Se_3_ nanostructures deposited on AAO and glass substrates prove the presence of the higher number of the tilted Bi_2_Se_3_ nanoplates growth on ordered AAO surfaces in comparison to the other substrates. As it can be seen from the XRD patterns (Fig. 4a,b), the dominating diffraction peaks of the Bi_2_Se_3_ grown on each of the considered substrates correspond to the growth of the nanostructures parallel to the substrate basis (peaks at 9.26, 18.58, 27.94, 37.66, 47.62 deg. correspond to the Bi_2_Se_3_ crystallographic planes 003, 006, 009, 0012, 0015 respectively).

**Figure 5:**
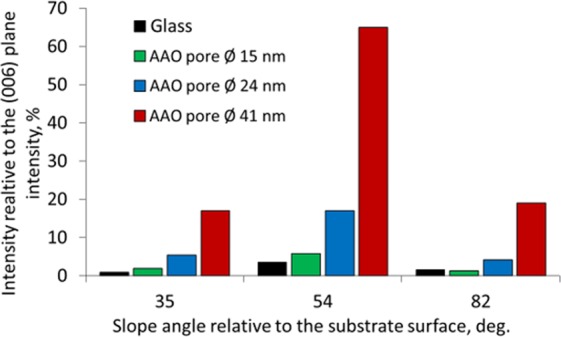
XRD peak intensities relative to the (006) plane intensity for different slope angles for growth of Bi_2_Se_3_ nanostructures on different substrates.

The lower intensity peaks at 40.30, 29.38 and 24.98 deg. correspond respectively to 1010, 015, and 101 crystallographic planes, tilted under angles 35, 54 and 84 deg. relative to the substrate basis. These tilt angles correlate with the slope angles of the nanopore openings retrieved from the SEM images of the AAO substrates (Fig. 4c–e). The intensity of all the peaks related to the tilted growth of Bi_2_Se_3_ on the substrate increases as follows: glass, AAO with disordered pores with diameter 15 nm, AAO with ordered pores with diameters 24 nm and 41 nm. These data are summarized in Table 1 and Fig. 5.

**Table 1:** XRD planes structure orientation relative to the substrate plane and intensity of XRD peaks for Bi_2_Se_3_ grown on different substrates.

XRD plane	Slope angle relative to the substrate surface, deg.	XRD intensity, counts			
		Glass	AAO disordered pores, diameter 15 nm	AAO ordered pores, diameter	
				24 nm nanopores	41 nm nanopores
1010	35	0.88	1.9	5.4	17.0
015	54	3.5	5.7	17.0	65.0
101	82	1.5	1.3	4.1	19.0

Insignificant differences between the XRD peak heights corresponding to the tilted Bi_2_Se_3_ nanostructures grown on the glass and the AAO surface with a disordered pore structure (pore size 15 nm) indicate that the vertical inner walls of the pores do not serve as nucleation centres for the growth of Bi_2_Se_3_ nanoplates. The intensities of the diffraction peaks for 35, 54 and 82 deg. angles for nanostructures grown on the AAO with ordered pore size of 41 nm are 3–4 times larger than these intensities for the nanostructures grown on the AAO surfaces with ordered pore size of 24 nm (Table 2, Figs 4b and 5).

**Table 2:** Yield and length of Bi_2_Se_3_ nanoribbons grown on different substrates.

Substrate	Density, nanoribbons/1000 μm^2^	Average length of 3 longest nanoribbons per 1000 μm^2^
Glass	2	30
AAO, disordered, 15 nm pores*	1	-***
AAO, ordered, 24 nm pores*	18	18
AAO, ordered, 41 nm pores*	23	19
AAO, ordered, 24 nm pores**	80	9

*Synthesis parameters: source material heating rate 12.5 °C/min, start-end pressure 5–20 Torr;

**Synthesis parameters: source material heating rate 18.5 °C/min, start-end pressure 0.5–4.5 Torr);

***Not enough nanoribbons to extract value, typical length of longest nanowire – 20–30 μm.

**Figure 6:**
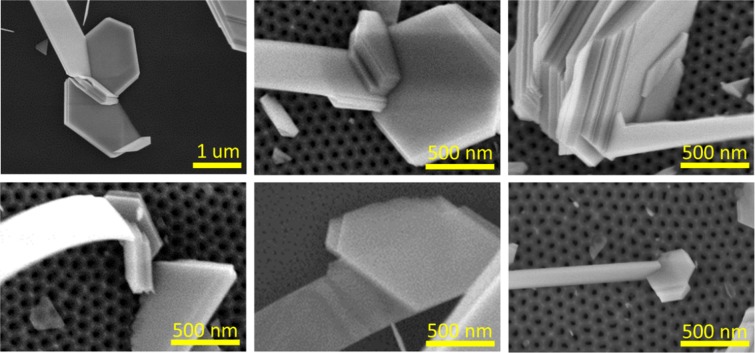
SEM images of the Bi_2_Se_3_ nanoribbons growing from the Bi_2_Se_3_ seed nanoplates.

Analysing the SEM images for all AAO substrates, there was no indications found on the growth of the nanoribbons directly from the AAO surface. The growth of all examined Bi_2_Se_3_ nanoribbons deposited on the AAO substrates was starting from the disordered nanoplate stackings or nanoplates of a non-hexagonal shape (Fig. 6).

Most of the nanoribbons start their growth from the side edges of the nanoplates. Data on the average number and length of the nanoribbons-per-unit-area grown on ordered and disordered AAO surfaces are presented in Table 2.

Table 2 shows, that the ordered AAO surface assures higher yield of the nanoribbons, which correlates with the increased number of tilted nanoplates. The number of the nanoribbons per unit area grown on the AAO surfaces with an average pore diameters of 24 nm and 41 nm increased by 18 and 23 times respectively in comparison to the number of the nanoribbons per unit area grown on the glass. Despite the 3–4 times difference in the XRD peak intensities related to the tilted nanoplates, the number of the nanoribbons on the AAO surfaces with pore size 41 nm is only 1.3 times larger than the number of nanoribbons on the surfaces with pore size 24 nm. Possibly, the tilted relative to the substrate surface nanoplates form more dense stacks on the AAO surfaces with pore diameter 41 nm, and the growth of the nanoribbons from the inner nanoplates in the stacks is obstructed.

Lengths of the nanoribbons in all samples varied from several micrometres up to 30 micrometres. The longest nanoribbons were synthesized on the glass substrate – the average length of 3 longest nanoribbons per 1000 μm^2^ is 30 μm. The average lengths for 3 longest nanoribbons grown on AAO with pore diameters of 24 nm and 41 nm are 18 and 19 micrometres respectively. Approximate width of nanoribbons varied between 100 and 600 nm for all substrates. The data on the nanoribbons widths are indicative as they were obtained from the SEM images, where only a projection of a nanoribbon is visualized, not allowing precise determination of the nanoribbon’s width.

The yield of the nanoribbons grown on the AAO surfaces with the average pore diameter of 24 nm can be increased by 80 times in comparison to the glass surface (Fig. 2b,d) by changing the synthesis parameters (the source material heating rate is increased by 6 °C/min with a simultaneously decreased base pressure down to 0.5 Torr, resulting in a decrease of the end pressure of the synthesis by ~15 Torr). However, the adjustment of the synthesis parameters for maximizing the yield of the nanoribbons results in a decrease of their average length down to 9 micrometres (Table 2).

### Magnetotransport properties

**Figure 7:**
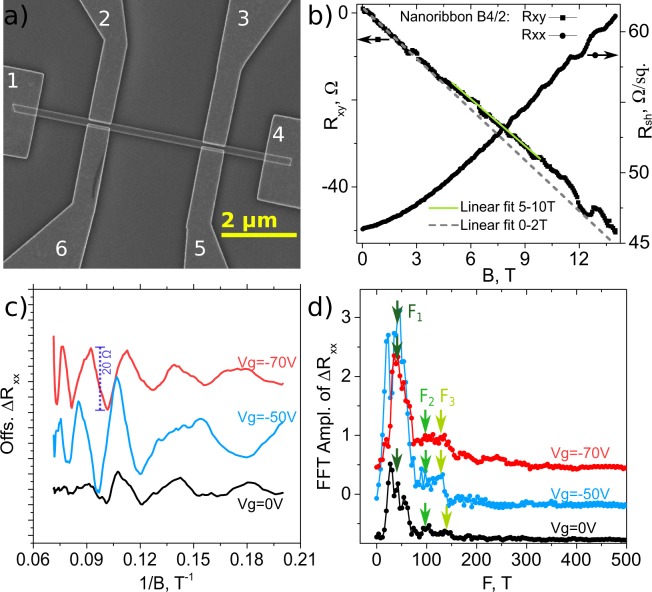
Magnetotransport measurements for Bi_2_Se_3_ nanoribbons: (**a**) – SEM image of individual Bi_2_Se_3_ nanoribbon Hall bar device; (**b**) – Magnetic field dependence of R_xy_, nanoribbon B4/2, t = 56 nm; (**c**) – Shubnikov-de Haas oscillations in longitudinal resistance ΔR_xx_ after subtracting a polynomial background, as a function of inverse magnetic field 1/B; (**d**) – Fast Fourier transform of Shubnikov-de Haas oscillations measured at 0 V, −50 V and −70 V back – gate voltage; nanoribbon A4.

Magnetotransport measurements were performed for the nanoribbons grown on the ordered AAO substrate with pore size 24 nm and the synthesis parameters: source material heating rate 18.5 °C/min, start-end pressure 0.5–4.5 Torr (Fig. 2d, Table 2). Figure 7a shows an image of one of the fabricated nanoribbon devices transferred to a Si/SiO_2_ substrate used for transport measurements. The longitudinal and transversal resistances R_xx_ and R_xy_ were measured simultaneously using electrodes 2–3 and 2–6 respectively, under a constant current flow between the probes 1–4. The magnetic field was applied parallel to the substrate surface normal, i.e. perpendicular to the nanoribbon axis.

The magnetic field dependence of the resistance R_xy_(B) is plotted in Fig. 7b. At magnetic fields above 2 T, the curve changes slope compared to the 0–2 T region (grey dashed line, Fig. 7b). This behaviour indicates multi-band transport as expected in topological insulators^3^.

The total density of the carriers can be extracted considering the highest magnetic field region in the R_xy_(B) dependence. At B > 10 T Shubnikov-de Haas oscillations in R_xx_^13,14^ (see R_xx_(B) in Fig. 7b) are typically detected. This can affect the R_xy_ magnetic field dependence and the region higher than 10 T cannot be used to extract the carrier density. Therefore the 5–10 T region of the R_xy_ (light green linear fitting curve in Fig. 7b of R_xy_) is used. The Hall carrier density *n*_3*D*_ for a nanoribbon is calculated from the Hall coefficient ${R}_{H}=t\frac{{\rm{d}}{R}_{xy}}{{\rm{d}}B}\times \frac{w}{{w}_{H}}=\frac{1}{{n}_{3D}q}$, where *w*_*H*_ is the distance between the transversal contacts, *w* is width of the nanoribbon, *t* is the thickness of the nanoribbon and *q* is the elementary charge^14^. The calculated carrier density can be used to extract the Hall mobility *μ*_*H*_ = 1/(*R*_*sh*_*n*_3*D*_ *t q*), where *R*_*sh*_ is the sheet resistance, estimated as *R*_*sh*_ = *R*_*xx*_*w*/*L*, here *L* is the length between the longitudinal electrodes (pairs 2–3 or 6–5, Fig. 7a). Table 3 lists all the calculated quantities for 4 nanoribbons.

**Table 3:** Physical parameters of Bi_2_Se_3_ nanoribbons grown on an AAO substrate with pore size of 24 nm and synthesis parameters: source material heating rate 18.5 °C/min, start-end pressure 0.5–4.5 Torr.

Nanoribbon	*t* (nm)	*R*_*sh*_ (Ω/sq.)	*n*_3*D*_ (1/cm^3^) × 10^18^	*μ*_*H*_ (cm^2^/Vs)
B5/3	33	151	16	780
A4	42	106		
A5	55	43	7.8	3410
B4/2	56	46	13	1920
ref.^29^	71	43	12	1721

The values of the Hall mobility, sheet resistance and 3D carrier density for the 56 nm thick nanoribbon B4/2 are close to previously reported values of the Bi_2_Se_3_ nanoribbons grown by gold particle catalysed vapour-liquid-solid synthesis^29^ (Table 3).

Figure 7c shows the oscillatory part of R_xx_ as a function of 1/B, after the removal of a smooth background, at a temperature of 2 K for various back-gate voltages (Vg = 0, −50 and −70 V). These are Shubnikov-de Haas (SdH) oscillations, periodic in 1/B. The fast Fourier transform (FFT) of ΔR_xx_ is given in Fig. 7d. The spectra at Vg = 0 V indicates one dominating frequency F_1_ = 43 T and two other low intensity frequencies F_2_ = 100 T and F_3_ = 147 T. When a large negative voltage is applied to the back gate (Vg = −50 and −70 V), the oscillations become more pronounced (Fig. 7c, blue and red curves) resulting in better resolved peaks in the FFT spectra (Fig. 7d). The dominating frequency F_1_ and one of the low intensity frequencies F_2_ do not change with the applied back gate voltage, while the largest frequency F_3_ is slightly moved to lower values from 147 T (Vg = 0 V) to 130 T (Vg = −50 V).

Previous studies on Bi_2_Se_3_ nanoribbons grown on glass substrates by a similar PVD approach as we report here, have also shown a multi-band transport, with carriers from topological surface states at the nanoribbon top surface, bulk carriers and trivial carriers from a 2DEG accumulation layer at the interface between the nanoribbon bottom surface and the substrate of Si/SiO_2_^14^. The accumulation layer possibly originates from the interface between the oxide surrounding the nanoribbon^14^ and the SiO_2_. In this work the nanoribbons are transferred to a similar type of Si/SiO_2_ substrate, so also in this case the presence of the accumulation layer at the interface of the nanoribbon bottom surface and the SiO_2_ can be assumed.

The bulk and the Dirac electrons at the top surface cannot be affected by back gate in a thick nanoribbon since they are electrostatically shielded by the bottom interface with the substrate^14^. The fact that the oscillation frequency F_3_ decreases with the back-gate voltage, therefore points to a depletion of the carriers at the interface with the substrate, which can be tuned by the back-gate voltage. In such a case, F_3_ could be attributed either to the trivial 2DEG (accumulation layer) or to the Dirac electrons at the nanoribbon bottom surface. In case of the Dirac electrons, the carrier density ${n}_{2D,BS}={k}_{F}^{2}/4\pi$, where ${k}_{F}$ is the Fermi wave vector, obtained by the relation $F=(\frac{\hbar }{2\pi e\,}){A}_{0},$ where ${A}_{0}=\pi {k}_{F}^{2}$ is the cross-section of the Fermi surface. The density *n*_2*D*,*BS*_ at Vg = 0 V would be 3.6 × 10^12^ cm^−2^.

The other two frequencies, F_1_ and F_2_ are instead constant with the back-gate voltage, therefore represent either the bulk carriers or carriers from the Dirac surface states at the nanoribbon top surface.

Considering that the nanoribbon is rather thick (t = 42 nm, nanoribbon A4, Table 3), it can be expected, that the carriers from the material bulk would yield the dominating frequency F_1_. However, from our measurements we cannot univocally attribute the oscillation frequency F_1_ to the bulk carriers. Therefore, in the following, we will discuss the two possible scenarios: 1) F_1_ is given by the bulk band and F_2_ is given by the top (topological) surface state; and 2) F_1_ and F_2_ are given by top (topological) surface states and the bulk band, respectively.

**Figure 8:**
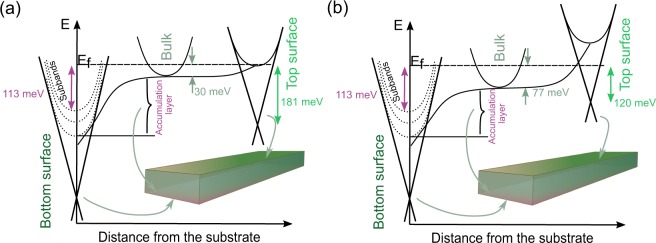
Schematics of the band-bending diagram for a Bi_2_Se_3_ nanoribbon on Si/SiO_2_ substrate: (**a**) Oscillation frequency F_1_ corresponding to bulk carriers; (**b**) Oscillation frequency F_2_ corresponding to bulk carriers.

For case (1) the bulk carrier concentration can be calculated as *n*_*B*_ = 1*/*(2*π*)^2^(4*/*3) *k*_*F*_^3^, and *n*_*B*_ = 1.6 × 10^18^ cm^−3^. The corresponding Fermi energy is given by ${E}_{F}^{B}={\hbar }^{2}/(2{m}^{\ast })\,{(3{\pi }^{2}{n}_{B})}^{2/3}$, where m^*^ is the effective mass of the Bi_2_Se_3_ bulk carriers. Assuming the mass m^*^ = 0.15 *m*_*e*_^30^, the Fermi energy of the bulk electrons is *E*_*F*_^*B*^ = 30 meV. With F_2_ to the top topological surface state we obtain *n*_2*D*,*TS*_ = 2.4 × 10^12^ cm^−2^. The corresponding Fermi energy (from Dirac point) is ${E}_{F}^{TS}=\hslash {k}_{F}{v}_{F}=181$ meV, where *v*_*F*_ is the Fermi velocity, *v*_*F*_ = 5 × 10^5^ m/s^2^. This implies an upward band bending of conduction band at the top surface of *∆E*_*BB*_^*TS*^ = *E*_*F*_^*B*^ − (*E*_*F*_^*TS*^ − 180 meV) = 29 meV^31^ (see Fig. 8(a)). These characteristics of the bulk and Dirac surface carriers are similar to our previous estimates for Bi_2_Se_3_ nanoribbons synthesized on a glass substrate^14^.

Attributing F_3_ to the bottom surface Dirac states we obtain *E*_*F*_^*BS*^ = 220 meV resulting in a downward band bending *∆E*_*BB*_^*BS*^ = −10 meV. The corresponding carrier concentrations of the bottom Dirac electrons together with the trivial electrons of the accumulation layer are 3.5 × 10^12^ cm^−2^ and 2.5 × 10^12^ cm^−2^, respectively. For the total carrier concentration we obtain *n*_2*D*,*Tot*_ = (*n*_2*D*,*TS*_ + *n*_*B*_ × *t* + *n*_2*D*,*BS*_) = 1.3 × 10^13^ cm^−2^, with *n*_2*D*,*BS*_ the total carrier concentration at the bottom interface. Comparing this value to the ${n}_{2D}^{Hall}$ values calculated from the Hall effect ~1 × 10^19^ cm^−3^ × *t* ≈ 4 × 10^13^ cm^−2^, it is clear, that attributing F_3_ to the bottom Dirac electrons cannot account for the larger value of the total carrier concentration extracted from the Hall measurements.

Instead, when attributing the oscillation frequency F_3_, previously assigned to the Dirac electrons from the nanoribbon bottom surface, to the carriers from the accumulation layer at the interface with the substrate we obtain ${n}_{2D,Int}={k}_{F}^{2}/2\pi$ = 7.1 × 10^12^ cm^−2^ with corresponding Fermi energy *E*_*F*_^*int*^ = 113 meV. This concentration value is about 4 times lower than what was found earlier^14^, which indicates that only those carriers from one of the sub-bands with a mobility high enough to satisfy μB ≫ 1 originate the SdH oscillations. The resulting band bending diagram is shown in Fig. 8(a). F_3_ and the carriers $({n}_{2D}^{Hall}-{n}_{2D,Tot})$ represent the interface carriers from the remaining subbands with lower mobilities.

For case (2) the bulk carrier concentration is given by *n*_*B*_ = 5.6 × 10^18^ cm^−3^ with corresponding Fermi energy *E*_*F*_^*B*^ = 77 meV. Similar to case 1), attributing the oscillation frequency F_3_ to the bottom Dirac states cannot account for the total carrier concentration extracted from Hall measurements. Instead identifying F_3_ to one of the trivial sub-bands at the interface we obtain the band bending diagram shown in Fig. 8(b).

## Conclusions

To conclude, the topological insulator Bi_2_Se_3_ nanoribbons were synthesized by catalyst-free physical vapour deposition technique on porous AAO templates. The density of the nanoribbons per unit area was found to be 20–100 times higher in comparison with the density of the nanoribbons per unit area synthesized onto glass substrates by the same method. It has been shown, that formation of the nanoribbons occurs mostly from the side facets of the seed nanoplates. Ordered porous AAO surfaces promote formation of tilted under different angles relative to the AAO substrate nanoplates due to the funnel type pore openings and the oxide asperities in the interpore space, which presumably are energetically favourable for adsorption of Bi and Se atoms and growth of Bi_2_Se_3_ nanostructures oriented in different directions.

Analysis of the back-gate dependent Shubnikov-de Haas oscillations shows carriers from three different populations– carriers from the Dirac surface states at the nanoribbon top surface, the bulk carriers and the carriers from a trivial accumulation layers at the nanoribbon and the substrate interface. Determined Hall mobility for individual Bi_2_Se_3_ nanoribbons grown on porous AAO substrates is rather high, which is beneficial for many applications and for fundamental studies.

## Methods

A standard two step anodization protocol was used for preparation of self-ordered AAO templates^32–34^. Ultrathin AAO membranes were synthesized by anodizing pure Al foil (99.999%, 0.32 mm, 10 × 18 mm, Good Fellow). The Al samples were degreased in acetone and electropolished in perchloric acid – ethanol mixture (1:4). The Al pieces were anodised in 0.3 M sulphuric acid electrolyte at a potential 25 V or 0.3 M oxalic acid solution at a potential 40 V. After 2 hours long anodization, anodic oxide layer was etched off using phosphoric acid/chromic acid mixture. AAO ultrathin films with approximate thicknesses of 250 nm (Fig. 1) were produced during the 60 seconds long second anodization under the same conditions as the first one. Thin disordered AAO templates were synthesized during 60 seconds long first anodization, using 0.3 M sulphuric acid solution at 25 V.

The catalyst-free vapour-solid synthesis method of Bi_2_Se_3_ nanoribbons synthesis was similar to the reported previously^13^. The synthesis was processed in a single-zone quartz furnace (GCL-1100X, MTI Corp). Bi_2_Se_3_ crystals (99.9% Sigma Aldrich) were used as source material and placed in the heated centre of the furnace tube^13^.

AAO substrates were placed inside the tube downstream from the source material, where the maximal temperature during the deposition process will achieve 350–400 °C. Before the synthesis, the furnace tube was flushed with N_2_ and pumped down to the base pressure 0.5–5 Torr and sealed. The source material evaporation temperature was heated up to 585 °C at rate 12.5–18.5 °C/min. When this temperature was achieved, the furnace was held at it for the next 15 minutes. At the end of this period, the source materials vapour pressure in the tube achieved 4.5–20 Torr; then the furnace was let to cool down naturally. The temporary gas flow (dynamic pressure ~ 25 Torr) was introduced in the furnace tube during the cool-down process for the temperature interval 535–475 °C for the first series of the experiments and 535–500 °C with the following pumping down, for the second series of the experiments. The gas flow was introduced to provoke formation of the nanoribbons. When the temperature of the furnace centre dropped down to 475 °C, the furnace tube was filled with N_2_ up to 800 Torr to terminate the growth of the nanostructures. The samples were taken out from the furnace after it cooled down to the room temperature.

Morphology and chemical composition of the synthesized Bi_2_Se_3_ nanostructures were investigated using field emission scanning electron microscope (SEM) Hitachi S-4800 equipped with an energy-dispersive X-ray (EDX) spectrometer Bruker XFLASH 5010. SEM images were analysed with ImageJ software^35^.

The crystalline structure of fabricated heterostructures was studied using X-ray diffraction spectroscopy (powder diffractometer X’PERT MRD with Cu Kα radiation source).

Bi_2_Se_3_ nanoribbons grown on AAO substrates were mechanically transferred to a n-type silicon substrate covered with 300 nm of SiO_2_. Optical imaging was used to select individual nanoribbons on the Si/SiO_2_ chip. Nanoribbon devices for magnetotransport measurements were fabricated by electron beam lithography. To achieve ohmic contacts, the Bi_2_Se_3_ nanoribbons were etched with Ar^+^ ions shortly before the metallization (Ti/Au). Magnetotransport measurements were performed in a Quantum Design Physical Property Measurement System (PPMS). All the data were recorded at base temperature of 2 K and in a four-probe configuration.
